# Standardised postprocessing of native T2 in detection and discrimination of myocarditis - comparison with native T1 mapping

**DOI:** 10.1186/1532-429X-18-S1-O14

**Published:** 2016-01-27

**Authors:** Rocio Hinojar, Lucy Foote, Ciara Cummins, David M Higgins, Eike Nagel, Valentina Puntmann

**Affiliations:** 1 grid.411088.40000000405788220Cardiology, University Hospital Frankfurt, Frankfurt am Main, Germany; 2 grid.13097.3c0000000123226764King's College London, London, UK; 3 Philips Healthcare, Guildford, UK

## Background

T2 mapping by cardiovascular magnetic resonance (CMR) has been recently proposed as a robust and accurate technique to identify the presence myocardial inflammation. Standardization of post-processing, including assessment of reproducibility and discrimination between health and disease, is necessary for implementation of this technique in clinical practice. Acute myocarditis is characterised by diffuse overspill of intracellular and interstitial edema and inflammatory infiltration. We examined regional T2 values in healthy volunteers (n = 40) and in patients presenting with clinical diagnosis of acute myocarditis (n = 59), and compared several postprocessing approaches to T1 mapping in discrimination between health and disease.

## Methods

All subjects underwent a CMR study for routine assessment of myocardialfunction and scar, as well as T1 and T2 mapping, using 3(3)3(3)5 MOLLI and GraSE sequence, respectively, using 3-Tesla clinical scanner. T2 maps were acquired in apical, midventricular and basal short-axis slice (SAX). Two independent observers measured T2 values from quantitative maps in 16 segments, complete SAX coverage and septal T2 values in patients and controls. Intra- and inter-observer reproducibility of T1 and T2 mapping was assessed using Bland-Altman methods. Discrimination between health and disease was assessed by binary logistic regression.

## Results

Intra- and inter-observer agreements for native T1 (r = 0.98; r = 0.97, p < 0.001) and native T2 (r = 0.97; mean difference (MD) ± SD = -0.05 ± 1.85; r = 0.95, p < 0.001 MD ± SD = 0.01 ± 2.5) values across the whole cohort were very high. Similarly, the intra- and inter-observer coefficients of variation (CoV) for T1 (0.66%; 1.19%) and T2 (1.85%; 2.5%) values were low. In healthy controls there were no significant difference between 16 segments (p = 0.21), SASX slices (p = 0.48) nor between segments and midventricular septal sampling (p = 0.67), inidcating no regional variations of T2 values in healthy myocardium.

Compared to controls, T2 values were significantly raised in patients with myocarditis (T2 values (msec: septal: 48 ± 3 vs. 56 ± 6, SAX: 49 ± 4 vs. 57 ± 8, p < 0.001). Septal T1 values were not significantly different to SAX T2 values of the same slice (r = 0.62, p < 0.001). Midventricular septal measurements were similar to segmental T2 values (p = 0.37). Midventricular septal (ConSept) T1 and T2 values were independent discriminators between health controls and patients with myocarditis. Native T1 values were more accurate than T2 values to (T1 vs. T2, diagnostic accuracy, 98 vs. 84%, (AUC T1: 0,99; T2: 0.89).

## Conclusions

We demonstrate that T2 values are useful in detection of myocardial inflammation, whereby septal T2 values are comparable to regional and SAX T2 values in healthy myocardium and acute myocarditis. Both native T1 and T2 using conservative myocardial septal measurements provide accurate, reproducible and easy to apply measurements of mycoardial inflammation.

**Figure 1:**
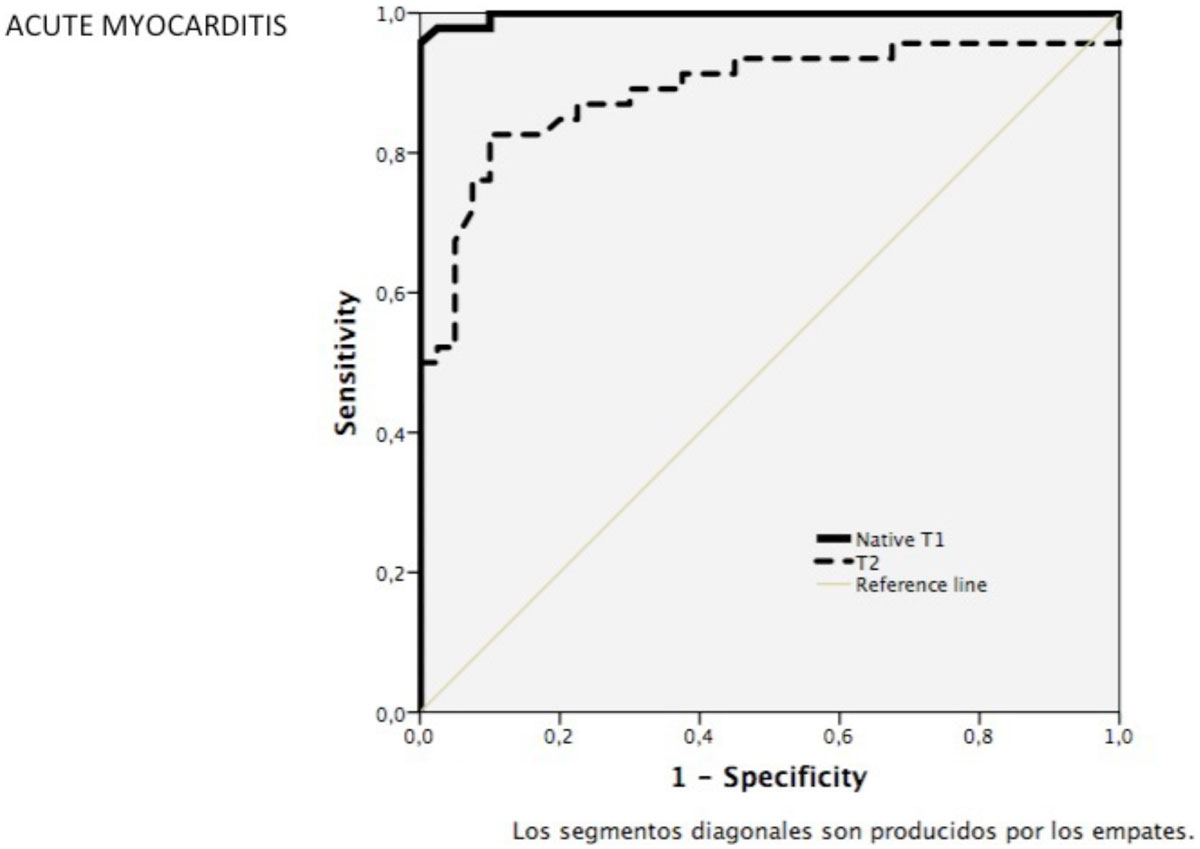
Figure 1

